# Effects of Thin Atmosphere

**Published:** 1884-08

**Authors:** 


### THE EFFECTS OF THIN ATMOSPHERE.

VIRGINIA City, Nevada, is a little more than 7,000 feet above sea level, yet even at that comparatively moderate altitude as compared with some other inhabited elevations, the housewife finds some difficulty in cooking by boiling, the water boiling at too low a temperature to thoroughly cook meat and vegetables. The *Virginia City Enterprise* says that there is complaint every year that the peas brought from California are as hard as buckshot. The trouble is that the water does not become sufficiently hot to cook them. Here, when either meat or vegetables are being cooked by boiling, the vessel used should have a close fitting lid, in order that the steam may be confined. There is, of course, no trouble about roasting meats or anything else, fire being as hot here as in any other part of the world. While strangers complain much of the thinness of our atmosphere, old settlers are not much distressed, and children born and reared here seem not to suffer inconvenience in any way. They race up and down the sides of the mountains at full speed without finding any difficulty in breathing.

